# Impaired large numerosity estimation and intact subitizing in developmental dyscalculia

**DOI:** 10.1371/journal.pone.0244578

**Published:** 2020-12-31

**Authors:** Gisella Decarli, Emanuela Paris, Chiara Tencati, Chiara Nardelli, Massimo Vescovi, Luca Surian, Manuela Piazza

**Affiliations:** 1 Department of Psychology and Cognitive Science, University of Trento, Rovereto, Italy; 2 Department of General Psychology, University of Padova, Padova, Italy; 3 Servizio di Logopedia, Azienda Pubblica di Servizi alla Persona “Beato de Tschiderer”, Trento, Italy; 4 Center for Mind/Brain Sciences—CIMeC, University of Trento, Rovereto, Italy; French National Center for Scientific Research (CNRS) & University of Lyon, FRANCE

## Abstract

It is believed that the approximate estimation of large sets and the exact quantification of small sets (subitizing) are supported by two different systems, the Approximate Number System (ANS) and Object Tracking System (OTS), respectively. It is a current matter of debate whether they are both impaired in developmental dyscalculia (DD), a specific learning disability in symbolic number processing and calculation. Here we tackled this question by asking 32 DD children and 32 controls to perform a series of tasks on visually presented sets, including exact enumeration of small sets as well as comparison of large, uncountable sets. In children with DD, we found poor sensitivity in processing large numerosities, but we failed to find impairments in the exact enumeration of sets within the subitizing range. We also observed deficits in visual short-term memory skills in children with dyscalculia that, however, did not account for their low ANS acuity. Taken together, these results point to a dissociation between quantification skills in dyscalculia, they highlight a link between DD and low ANS acuity and provide support for the notion that DD is a multifaceted disability that covers multiple cognitive skills.

## Introduction

Humans possess two basic non-verbal systems underlying the quantification of the number of objects in sets. One system allows approximate estimation (also called approximate number system, or ANS) and it generally follows the Weber law, according to which the threshold of number discrimination between two stimuli increases proportionally with the intensity of the stimuli, i.e. with the magnitude of the numerosities. More recently, some authors have proposed that the discrimination threshold follows the Weber law only in the case of low-density numerosities, while for high-density and crowded numerosities a texture-like mechanism drives the numerosity comparison [1–3] (i.e., when the dots are too dense, numerosity judgements seem to be performed using a texture-based mechanism, evident from the fact that in those cases number discrimination threshold increases with the square root of numerosity instead of with its log). The other numerical system allows exact and fast quantification but it is limited in capacity, as it only applies to small sets of few objects (also called subitizing, or object tracking system, OTS).

The distinction between these two systems [4–9], evident in human adults [10, 11], is traceable from the very first days of life. Young infants, able to discriminate large sets differing by at least of 200% (e.g., 10 *vs*. 30 items), are not able to distinguish sets differing by the same amount in the small number range (e.g., 1 *vs*. 3), as their still immature multiple object tracking system only extends to sets of 1–2 objects [6]. According to some authors, young infants present an immature OTS, that would develop during the first year of life [12, 13]. As demonstrated by some studies that have assessed the visual short-term memory (the ability at the base of the tracking system), infants present a span of one object at 6 months, and it develops up to 4 elements at 12 months.

Both systems improve during the lifespan (due to maturation and experience), but their developmental trajectories appear rather different: while the multiple object individuation ability reaches the adult level during early childhood, the approximate number system continues to improve up to adulthood. Moreover, there is some evidence that small sets individuation and large sets quantification are dealt with by separate cognitive and neuronal mechanisms through the entire life-span [8, 14, 15]. The qualitative difference between these two systems appear evident when looking at neuroimaging data, since each system appears associated to specific alteration of the EEG signal, in both adults and preverbal infant: the OTS corresponds to the modulation of an early posterior brain wave, which amplitude varies proportionally with number, but only for small sets of 1–3 items; the ANS, on the contrary, gives rise to a ratio-dependent modulation of a late posterior wave only during processing of large numerosities [14]. This suggests that the ANS and OTS may emerge from at least partially segregated cortical circuits. More recently, Fornaciai and Park [15] identified distinct polarity as well as topographic distributions of electrodes sensitive to small and large numerosities specifically.

One aspect that further differentiates these two systems is the extent to which they correlate and longitudinally predict formal mathematical skills. On one side there is now solid evidence for a reliable and bidirectional link between the ANS acuity (the precision in assessing the number of items in large sets) and formal math skills: the ANS correlates and longitudinally predicts mathematical skills [16–19], and is in turn modified by the acquisition of formal math skills [20, 21]. One potential interpretation of these results is that when children learn the meaning of symbols they initially do so by relating them to the pre-existing representations of non-symbolic magnitudes and their transformations, and that the two systems remain connected during the life-span [22; however see 23 for a different view]. For example, Gilmore and colleagues showed that 5-to-6-year-old children during the first stages of acquisition of symbolic numbers treat them as symbolic referents to approximate quantities [24]. Traces of this link can also be found in older children and in adults who, when asked to compare symbolic numbers, show the same distance and magnitude effects (i.e., speed and accuracy decrease as the ratio across numbers increases) that also characterize non-symbolic magnitude processing. However, it should also be noted that other authors failed to find an association between symbolic and non-symbolic formats. For example, some authors have found higher RTs and error rates in comparing mixed format of dots-numerals, than single format of dots-dots, concluding that if the symbolic and non-symbolic formats (one highly accurate, while the other highly inaccurate) are linked then the performances should not be worse in the mixed format compared to the single one [25]. Moreover, some neuroimaging findings failed to provide evidence for a common neuronal representation between the two formats [e.g., 26]. In sum, the issue is still heavily debated.

On the other hand, as for the OTS, the rare studies focusing on the inter-individual differences in exact small number processing (typically indexed by the range of numerosities enumerated with no errors and in a very short time, also referred to as “subitizing range”) fail to find a reliable association with math abilities in typically developing populations [27].

These observations suggest that large number estimation and small number quantification might also be differentially involved in developmental dyscalculia (DD), a neurodevelopmental specific learning disability in number and calculation skills. Children with DD show a broad range of deficits in math that primarily include mental and written calculation but also extend to more basic skills such as number comparison or numbers to magnitudes association. Previous studies also reported a low ANS acuity in dyscalculia, indexed by impairments in dot comparison tasks, where DD seem to have a 5 year-delay along the typical developmental trajectory [28–32]. These findings, however, are not undisputed, since using similar (but not identical) dots comparison tasks, some researchers did not find differences between DD and controls [33–35]. Moreover, there is an ongoing debate on the factors underlying the DD poor performance in this task: some reported evidence that it is determined by a non-specific deficit in a general inhibitory system [36], while others failed at replicating it [37].

The literature on small set quantification (subitizing) in DD appears at least as equally controversial, if not more. Again, some authors found impairments in DD [38–42] while others did not [43–45].

Of particular interest for the question of whether the estimation and subitizing are or not commonly impaired in dyscalculia are those studies that investigate the two skills in the same group of DD children. These types of studies report divergent results: one did not find any deficit in any kind of set quantification (neither in subitizing nor in estimation) [46]; another found impaired estimation but not in subitizing [47], and a final one found an impairment in both estimation and subitizing [48]. To our knowledge no study to date reported an impairment in subitizing but no impairment in estimation in DD, potentially suggesting that whenever quantification tasks are compromised, subitizing tends to be more resilient to impairments compared to large set estimation.

When considering the results on subitizing, some methodological considerations are worth mentioning: first, many of the aforementioned studies investigated subitizing using a task where the to-be-enumerated dots remained on the screen for an unlimited time until the response of the participant was detected [38, 41, 42, 46–48]. Giving no limits to stimuli presentation could lead children with DD, who tend to be more unsure whenever they have to deal with numerical tasks, to adopt a different strategic choice compared to controls, such that of counting the dots one by one also when presented with sets of 1–3 objects. This would be coherent with the fact that the aforementioned studies reported an impairment in RTs (some in the form of a steeper increase in RTs) but not in accuracy in DD. This possibility reminds us that, while very often response times give important information on the structure of the internal representations, they may also be heavily influenced by strategic choices.

A second methodological concern is the definition of subitizing range. In some previous studies the interval defined as the subitizing range was set not on the bases of the data, but a priori (to either to 3 or 4) [41, 45–47, 49]. However, it is known that the subitizing range is influenced by specific features of the paradigm, such as presentation time, the spatial layout of the stimuli, the presence of a mask [50, 51] and also the age range of participants [52]. Therefore, the subitizing range should be determined empirically on the data and not a priori. This might seem like a minor aspect. However, we believe it is not. In fact, setting the subitizing range a priori may lead to the inclusion of data that, given the particular protocol used, already fall in the serial counting or estimation regime instead of subitizing properly. This might thus let the researchers conclude that the DD have an impaired subitizing range when in fact they are impaired in serial counting, a process that, contrary to subitizing, involves serial visuo-spatial attentional shifts, working memory, and phonological processing [53].

In sum, while there is not full consensus as for whether dyscalculia is associated in deficit in large sets estimation, the question of whether they are impaired in subitizing is even more controversial. Particularly problematic in this respect seems the large variety of protocols used to investigate subitizing together with often arbitrary assumptions related to the extent of the subitizing range. The absence of a clear convergence on the issue of whether children with DD are impaired in both number estimation and subitizing clearly calls for further investigations.

In the present study we assessed numerosity estimation and exact enumeration performances, within the same group of children with developmental dyscalculia, and we compared them with those of non-dyscalculic children of the same age and general intelligence. Because the tasks typically used to assess numerosity perception rely on visually presented sets of items, one may think that potential impairments in these tasks could be directly depending on domain-general impairments in visuo-spatial skills. Several authors have indeed provided evidence that DD is associated with deficits in visuo-spatial skills, especially in visuo-spatial working memory [54–57]. In order to investigate the potential dependency between number perception and visuo-spatial skills we also tested the latter, using both a perceptual change detection task as well as the widely used Corsi working memory test.

The aim of the present study was to investigate whether children with DD are concurrently impaired in numerical judgements of large uncountable sets as well as small countable ones, tapping respectively on the ANS and on the OTS system. In parallel, we also assessed visuo-spatial short-term memory, a domain-general skill that, on the bases of previous research, we hypothesized could have a link especially with the OTS system. Indeed, previous studies, using correlational and dual task interference paradigms on adult subjects, demonstrated not only a dissociation between ANS and subitizing, but also a correlation and a specific interference between subitizing (but not the ANS) and visuo-spatial attention [4; 10; see also 11], suggesting that the OTS (but not the ANS) share resources with the visuo-spatial attention system.

We therefore hypothesized that dyscalculics would be impaired in the ANS and that this impairment would not correlate with visuo-spatial working memory skills. On the contrary, based on the aforementioned literature that points towards a link between OTS and visuo-spatial skills, we predicted that if the subitizing is impaired in DD, the impairment would be accompanied with concurrent impairments in visuo-spatial attention.

## Materials and methods

The research has been approved by the Human Research Ethics Committee of the University of Trento (approval number 2015–026). Consensus for participation in the study was written and was obtained from the parents of the children.

### Participants

The study included thirty-two dyscalculic children and thirty-two typically developing children with no learning disabilities. Parents/legal representatives gave written consent to participate in the study. The experiment was performed in accordance with the ethical standards established by the Declaration of Helsinki.

#### Children with DD

Thirty-two children with severe deficits in the mathematical domain were selected from a sample of children referred to a regional reference center for the diagnosis and rehabilitation of learning disabilities, the “Azienda Sanitaria Beato de Tschiderer” (Trento, Italy), because of a specific learning disability in the mathematical domain. Their mathematical abilities were assessed through one of the following widely used age-standardized Italian batteries for assessing mathematical skills: (1) the “Battery for the assessment of Developmental Dyscalculia” (BDE-2) [58], a standardized battery for children aged 8 to 13 years which items are divided into 3 areas: number reading/writing, exact oral/written calculation and semantic number understanding (comprising symbolic number comparison, number-to line mapping, and approximate calculation), or (2) the “Test for evaluating Calculation Abilities” (AC-MT) [59], a standardized battery for children aged 6 to 11 which produces 8 indices that consider speed and/or accuracy in written and mental calculation, verbal reciting of the number list, number spelling (dictation) and arithmetic facts retrieval. Both batteries provide normative data for every school grade; the raw score of each subtest is compared with the corresponding normative score to obtain the level of the child performance compared to typically developing children with the same age and grade. Therefore, each child performance can be assessed in terms of percentiles for the AC-MT and quotients for BDE-2.

Participants, included in the sample, presented severe difficulties in number processing and calculation corresponding to the strict diagnostic criteria for DD: performances under or equal to the 5th percentile in at least 4 out of 8 indexes of the AC-MT (N = 18) or in at least 2 out of 4 quotients of the BDE-2 (N = 14). We also included 1 subject who did not perform the full AC-MT battery, but only completed 2 out of 5 tasks: the mental calculation and the arithmetic facts retrieval tasks. In both tasks, and for both RTs and accuracy, he performed below the 5th percentile. An additional subject was included even if performance fell below the 5th percentile only in 2 (instead of 3 indices), but was consistently below the 10th percentile in 4 additional ones.

Further selection criteria were: general intelligence, assessed with the Wechsler Intelligence Scale for Children [60], within the normal range; normal or corrected-to-normal vision and hearing; normal schooling; no neurological or psychiatric disorders. Co-morbidity with other developmental disorders was not set as an exclusion criterion because our aim was not to generally describe the cognitive profiles of “pure” DD, but rather to contrast performance in two specific quantification tasks in children with DD, irrespective of the presence of other concurrent weaknesses. In our group, 14 children presented a selective deficit in the domain of mathematics (they were “pure” DD), while the remaining ones presented co-occurring deficits in other cognitive domains (written expression (N = 3), reading (N = 3), written expression and reading (N = 7), language production (N = 5), motor coordination (N = 1), or attention-deficit/hyperactivity (N = 1).

The group had a mean age of 9.51 (*SD* = 1.55, range 7.4–14) and an average total IQ of 97 (*SD* = 9.48) (Verbal IQ = 108, *SD* = 12; Performance IQ = 102, *SD* = 11). Children were tested in a quiet room of the "Azienda Sanitaria Beato de Tschiderer", in one or two sessions (separated by a max. of 14 days), depending on their availability. The order of the tests was randomized across participants.

#### Non-dyscalculic controls

Thirty-two children (mean age = 9.79, *SD* = 1.66, range 7.5–14.3) were selected from a sample of eighty-seven children, recommended as normal calculators by their teachers, from a primary school in Malo (province of Vicenza, in the north of Italy) to match the DD group in chronological age (Age: *t*(62) = -.71, *p* = .48), and in general intellectual functioning (Similarities, taken as an index of verbal skills, and Matrix Reasoning indexing visual processing. Note that 3 DD children were addressed to the Center after they were already diagnosed elsewhere, and for them we did not have the WISC single scores for Similarities and Matrix Reasoning, but only the total scores) (Similarities: *M*_*dysc*_ = 10.55, *SD* = 2.9; *M*_*contr*_ = 10.34, *SD* = 2.39, *t*(59) = .31, *p* = .76; Matrix Reasoning: *M*_*dysc*_ = 9.1, *SD* = 2.51; *M*_*contr*_ = 10.03, *SD* = 2.52, *t*(59) = -1.44, *p* = .16; see Table 1). The typically developing children were tested in a quiet room of the school, during school-time, in one test session. Also in this case, the order of the tests was randomized across participants.

**Table 1:** Mean and standard deviations for both groups in age and IQ.

	Age	Similarities	Matrix Reasoning
	*M (SD)*	*M (SD)*	*M (SD)*
**Dyscalculics**	9.51 (1.55)	10.55 (2.9)	9.1 (2.51)
**Controls**	9.79 (1.66)	10.34 (2.39)	10.03 (2.52)

### Tasks and procedure

#### Symbolic number comparison task

Because our control group did not perform the diagnostic tests for dyscalculia, we wanted to have to least one measure of symbolic number processing that would confirm their difference with the DD group. We therefore used a classic symbolic number comparison task. Participants were presented with pairs of stimuli, appearing within white circles on a computer screen. Stimuli consisted in Arabic digits from 1 to 9. Participants were asked to decide as quickly as possible which of the two digits indicated the larger quantity. Stimuli remained on the screen until participants pressed a response-key on the mouse. The task started with 8 training trials where feedback on accuracy was given to participants, and then it comprised 128 trials divided into 8 blocks, where no feedback was provided. It lasted about 10 minutes.

#### Large numerosity comparison task

**Fig 1:**
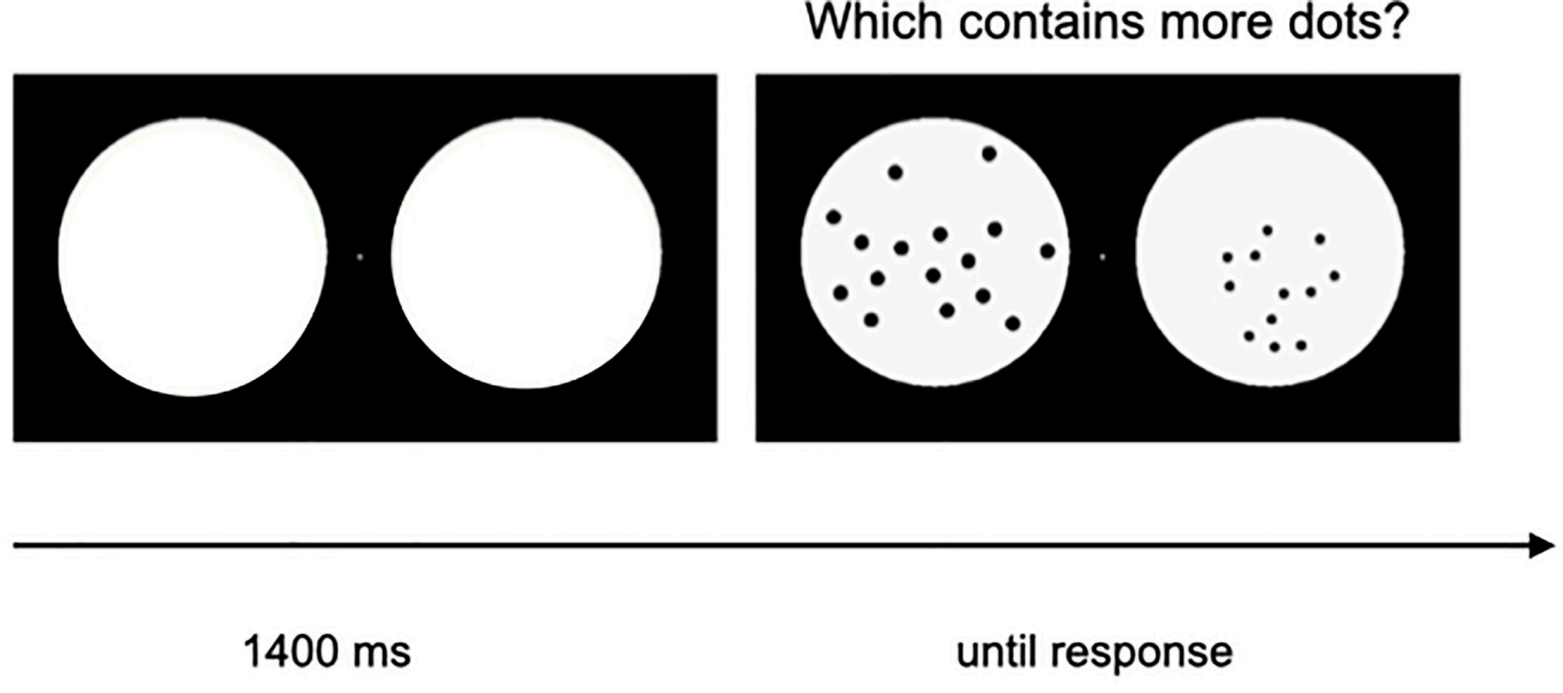
Example of stimuli used in the non-symbolic dot comparison task.

Participants were presented with pairs of arrays of black dots on white background, appearing laterally on a central fixation cross on the screen (Fig 1).

They were instructed to judge, fast and without counting, the more numerous one, by pressing the response-key on the mouse located on the same side. The arrays remained on the screen until children gave the answer.

Of each pair of sets, one (n1) always contained either of 16 or 32 dots. Stimuli paired with arrays of 16 dots (n2) could contain 12, 13, 14, 15, 17, 18, 19, 20 dots, while stimuli paired with 32 dots could present 24, 26, 28, 30, 34, 36, 38 or 40 dots. In half of the trials the size of the dots remained constant across all the n1 and n2 sets (therefore, as a consequence, the total occupied area increased with number), while for the other half the size of the total occupied area remained constant for the two arrays (thus individual item size was anti-correlated with number). The task started with 8 training trials where feedback on accuracy was given to participants, followed by 128 experimental trials, divided into 8 blocks, where no feedback was provided. It lasted about 10 minutes.

#### Small exact enumeration task

**Fig 2:**
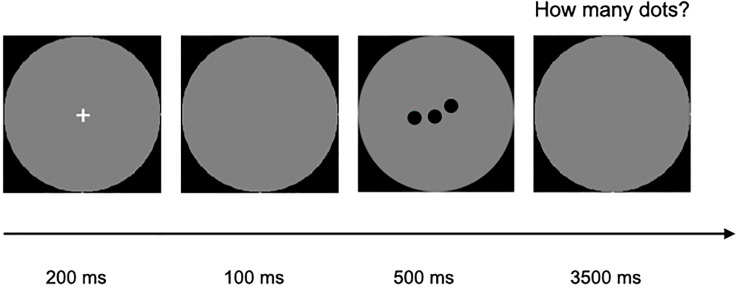
Exact enumeration task: Schema of timing and structure.

Participants were presented with arrays of colored dots varying in number from 1 to 8, and appearing, for 500 ms in a central grey circle (Fig 2).

Every dot had a specific color that was randomly chosen among 8 highly discriminable colors (red, pink, yellow, orange, blue, white, green, cyan and black) so that two dots could not have the same color in the same image. The stimuli were identical to those used for the visuo-spatial short-term memory task [see 10]. Participants were asked to say aloud and as quickly and accurately as possible the precise number. They were informed that the maximum number of dots could be 8. The responses were recorded via a microphone mounted on headphones to maintain the stability of the mouth-to-microphone distance across trials. In order to calibrate the microphone’s sensitivity to the average children’s voice pitch, before starting the task participants were instructed to say aloud numbers from one to eight. The microphone level was then calibrated accordingly. Stimuli were generated such that, across numerosities, half were controlled for size and half for total occupied area. The task comprised 10 training trials, followed by 128 trials divided into 4 blocks, and lasted about 30 minutes. No feedback was provided.

#### Visuo-spatial short-term memory task

Participants were presented with a sequence of two stimuli, both appearing in the center of the screen. Both stimuli were sets containing the same number of colored dots. In half of the trials, the second set was identical to the first one. In the other half, one of the dots appeared in a different color. The first image appeared for 700 ms, followed by a blank image for 1000 ms. Then, the second image remained on the screen for 2000 ms. Stimuli were identical to those used in the exact enumeration task. The task closely matches those typically used to assess visuo-spatial short-term memory in experimental psychology/psychophysics [10, 61].

Participants indicated whether the two arrays were identical or differed by expressing their judgment aloud, and an experimenter pressed the corresponding answer on the keyboard. While typically adult subjects perform this task through button press following simple response mapping rules (e.g., press right if same, left if different), here we used the vocal response to rule out that our results might reflect a potential difficulty in maintaining in memory the mapping rule. The experiment started with 10 training trials, followed by 128 test trials divided into 4 blocks. No feedback was provided.

#### Corsi block-tapping task “forward” and “backward”

The canonical version of the Corsi test was used [62]. The stimulus consisted of nine blocks placed on a board. The blocks were numbered from 1 to 9 only on one side of the block, visible to the experimenter but not to participants. The experimenter, seated in front of the child, taps the blocks with the finger following a sequence. Participants were instructed to repeat the same sequence with their own fingers. The task started with a sequence of two blocks, and then the experimenter increased in the number of blocks when two out of three trials of the same number were performed correctly. Otherwise, the test was terminated. In the “backward” version participants had to repeat the sequences in inverse order.

## Results

### Symbolic number comparison task

We analyzed mean accuracy and mean RTs using a mixed ANOVA with group (dyscalculics and controls) and numerical distance (1–8: e.g., pair 1–3, numerical distance = 2; pair 2–9, numerical distance = 7) as between and within factor respectively. Results indicated that dyscalculic children made significantly more errors and were slower than controls (main effect of group for accuracy: *M*_*contr*_ = .97, *SD* = .02, *M*_*dysc*_ = .95, *SD* = .03; *F*(1,62) = 8.94, *p* = .004, η^2^_G_ = .04; for RTs: *M*_*contr*_ = 920.04 ms, *SD* = 238.26 ms; *M*_*dysc*_ = 1139.2 ms, *SD* = 321.49 ms; *F*(1,62) = 10.07, *p* = .002, η^2^_G_ = .12), and that, for both groups, close numbers were more difficult to compare than distant numbers (main effect of distance for accuracy: *F*(7,434) = 57.04, *p* < .001, η^2^_G_ = .4; for RTs: *F*(7,434) = 53.7, *p* < .001, η^2^_G_ = .1). Importantly, however, dyscalculics were more impaired at comparing pairs of numbers characterized by a small numerical distance, indicated by a significant group by distance interaction for RTs and by a marginally significant interaction for accuracy (accuracy: *F*(7,434) = 1.87, *p* = .07, η^2^_G_ = .02; for RTs: *F*(7,434) = 2.48, *p* = .02, η^2^_G_ = .005).

### Large numerosity comparison task

We first analyzed overall mean accuracy: controls were significantly more accurate than DD (*M*_*contr*_ = 67.77%, *SD* = 6.59; *M*_*dysc*_ = 63.84%, *SD* = 7.8, *t*(62) = -2.18, *p* = .03, *d* = .54). Importantly, the two groups did not differ in RTs (*M*_*contr*_ = 1595.79 ms, *SD* = 632.45; *M*_*dysc*_ = 1556.38 ms, *SD* = 624.91, *t*(62) = -.25, *p* = .8, *d* = .06); thus, the poorer performance of DD children was not simply the result of an overall faster decision making.

**Fig 3:**
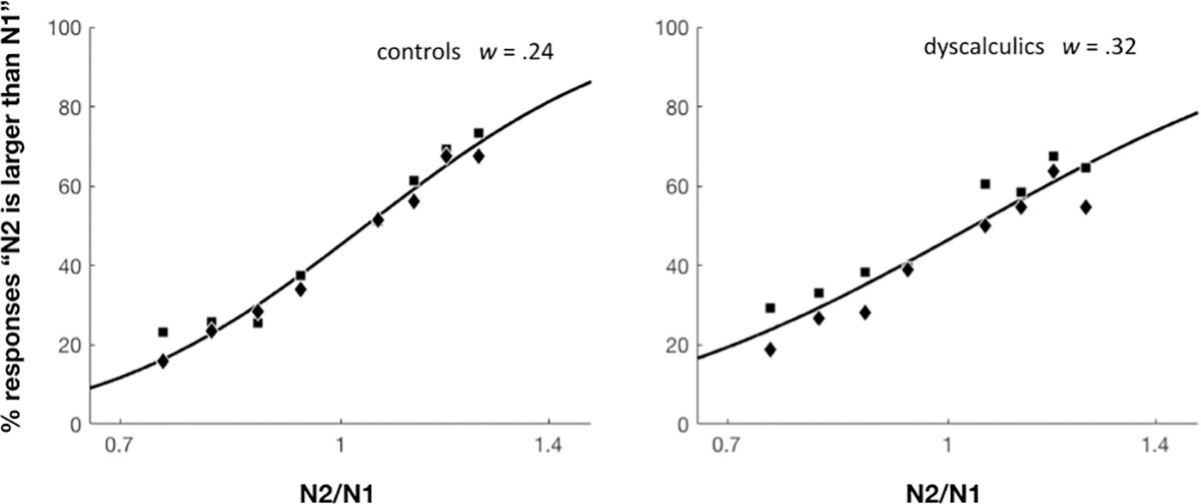
Large numerosity task: Weber fraction in children with DD and controls. Psychometric functions relating the percentage of trials in which N2 was reported as more numerous than N1 as a function of the logarithm of the ratio between N2 and N1. Squares N1 = 16; diamonds N1 = 32. The fitted curves are derived from the equations described in Piazza *et al*. [63].

In order to directly compare the current results with previous reports, we also analyzed the psychometric curves and estimated the internal Weber fraction (hereafter *w*) for each participant [32]. Assuming the hypothesis that numerosities are internally represented by a logarithmic number line with fixed Gaussian variability, *w* is proportional to the standard deviation of the estimated Gaussian distribution of the internal numerical representation that generates the observed performance. Because this measure is dependent upon model fitting, we excluded the subjects for which the model did not fit well (R2 < 0.2; N = 6, 4 dyscalculics and 2 controls). After this selection the two groups remained balanced for IQ and age (Similarities: *p* = .69; Matrix Reasoning: *p* = .32; age: *p* = .49). The DD group had larger *w* fractions (*M*_*contr*_ = .24, *SD* = .10; *M*_*dysc*_ = .32, *SD* = .14; *t*(55) = 2.55, *p* = .01, *d* = .68; see Fig 3).

RTs also did not differ across groups in these reduced samples (RTs: *M*_*contr*_ = 1645.39 ms, *SD* = 644.63 ms; *M*_*dysc*_ = 1622.37 ms, *SD* = 631.22 ms, *p* = .89, *d* = .04).

### Small exact enumeration task

**Fig 4:**
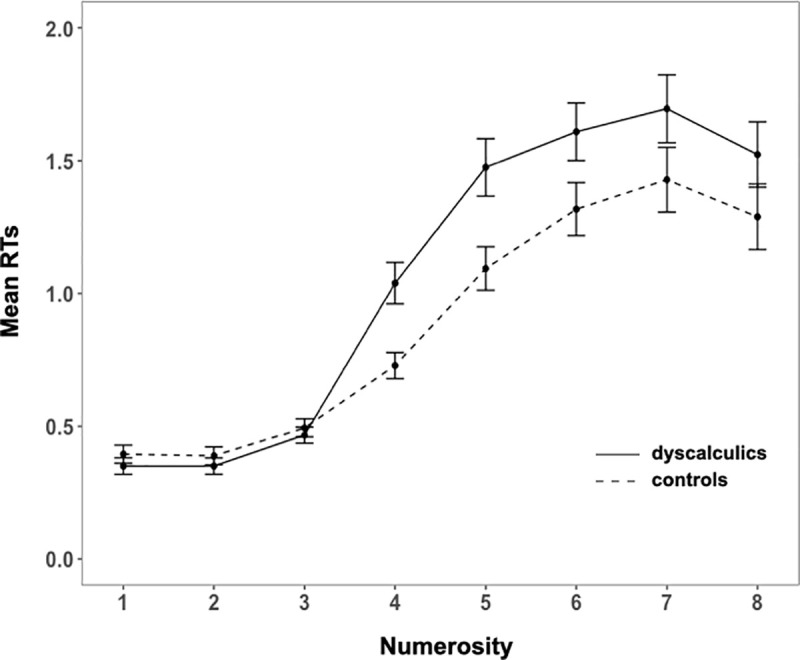
Exact enumeration task: Mean RTs in DD and controls. Error bars represent the standard error of the mean.

**Fig 5:**
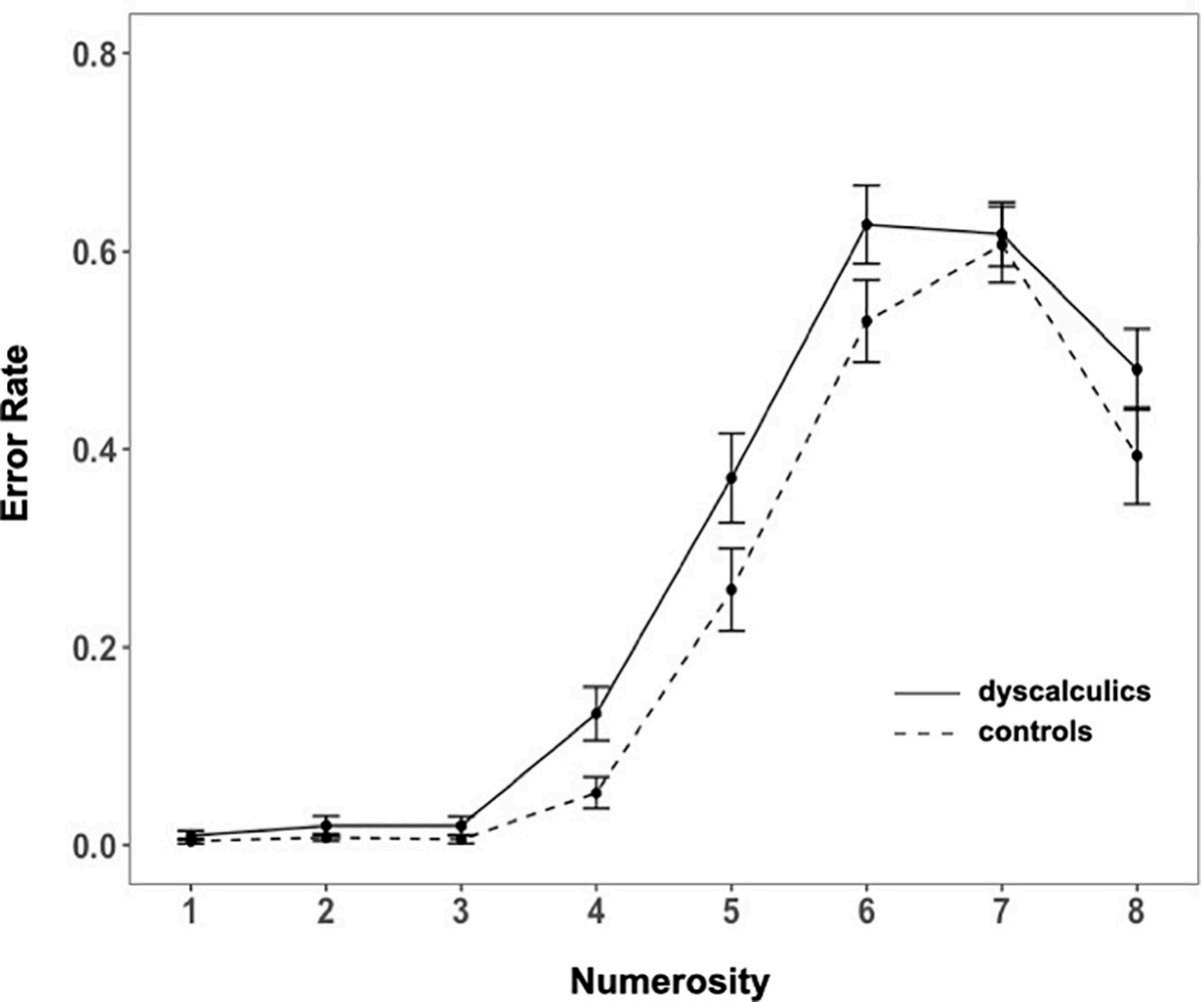
Exact enumeration task: Mean error rate for DD and controls. Error bars represent the standard error of the mean.

We first analyzed mean RTs and mean accuracy (Figs 4 and 5, respectively) through a mixed ANOVA with group as between factor and numerosity as within factor.

We reported the analyses including the set sizes up to numerosity 7, because children were informed that the number of dots presented could be from 1 to 8. Therefore, the results of numerosity 8 were influenced by this information given to the participants. Indeed, for numerosity 8 we found a typical guessing end-effect [see 10, 64, 65], showing a drop in RTs and in error rate compared to numerosity 7. The ANOVA revealed that both errors and RTs increased with number (main effect of numerosity accuracy: (*F*(6, 372) = 251.7, *p* < .001, η^2^_G_ = .72; RTs: *F*(6, 372) = 144.84, *p* < .001, η^2^_G_ = .55), and that dyscalculics made more errors and were slower (main effect of group accuracy: *F*(1, 62) = 4.1, *p* = .05, η^2^_G_ = .02; RTs: *F*(1, 62) = 4.6, *p* = .04, η^2^_G_ = .03; interaction for accuracy: *F*(6, 372) = 1.94, *p* = .07, η^2^_G_ = .02); interaction for RTs: *F*(6, 372) = 4.79, *p* < .001, η^2^_G_ = .04).

In order to specifically investigate whether dyscalculics present a reduced subitizing range we first estimated it by conducting pairwise comparisons among successive numerosities (1 *vs*. 2; 2 *vs*. 3; 3 *vs*. 4) on both accuracy and RTs in our control group. Since this is a study testing whether a clinical group is different from a control group, we estimated the “standard behavior” from the control group and then test if the clinical group differs from it or not. However, while in theory we could have also decided to perform the opposite analysis (set the standard subitizing range in the DD and then test if the controls behave differently), this would have been a much less sensitive approach for detecting a difference across groups: if, as we might expect from previous literature, dyscalculics have a smaller subitizing range than controls [e.g., 39], then the subitizing range computed from DD would include that of the controls. This would lead to no difference across groups, and to an erroneous conclusion. We found that significant differences in both accuracy and RTs appeared only between 3 and 4 (accuracy: 3 *vs*. 4: *p* < .01, all previous pairwise comparisons were not significant; RTs: 3 *vs*. 4: *p* < .001, all previous pairwise comparisons were not significant), suggesting that on this task our control group displays a subitizing range of 1–3. We thus proceeded performing separate analyses for the trials in the 1–3 (subitizing) and 4–7 (counting) range. Across groups, in the subitizing range there were no significant differences neither in accuracy (*M*_*contr*_ = 99%, *M*_*dysc*_ = 98%; *t*(62) = -1.3, *p* = .197, *d* = .33) nor in RTs (*M*_*contr*_ = .42 s, *M*_*dysc*_ = .39 s; *t*(62) = -.85, *p* = .4, *d* = .21). However, the groups did perform differently in the counting range, as shown both in their RTs and, only marginally, in accuracy (RTs: *M*_*contr*_ = 1.05 s, *M*_*dysc*_ = 1.36 s; *t*(62) = 2.71, *p* < .01, *d* = .68; accuracy: *M*_*contr*_ = 64%, *M*_*dysc*_ = 56%; *t*(62) = -1.94, *p* = .06, *d* = .49).

Another possible approach previously used to estimate the subitizing range is that of fitting a sigmoid function to the data and of taking its flex as its estimate [10, 11]. However, the sigmoid function is fitted on the full data set, including responses to both small and large numerosities. Because of this, the estimated flex is determined by how the data changes within the small and the large numerosity ranges: for any fixed performance in the small numerosity range, the steepness of the increase in performance within the counting range influences the estimate of the flex. Because DD and controls differed in both RTs and errors in the large numerosity range, this would have impacted on the estimate of the flex. On the contrary, computing pairwise comparisons between neighboring small numerosities is not influenced by performance with higher numerosities. For this reason, we have decided to use the first index instead of the flex.

We decided to perform two additional analyses to provide further evidence at the claiming of no difference between DD and controls in the subitizing range. We chose two indexes that had already been used in the literature when assessing children’s ability in enumerating small numerosities. The first one consists in combining RTs and accuracy in the so-called inverse efficiency measure (EM = mean correct reaction times / accuracy) and computing the steepness of the increase of EM (EM maxN—EM minN / EM maxN) within subitizing and counting ranges separately [66]. We compared those indices across groups, and we found no effect in the subitizing range, and a small but not significant difference in the counting range (subitizing range: *M*_*contr*_ = .2, *SD* = .23, *M*_*dysc*_ = .26, *SD* = .24; *t*(62) = 1.01, *p* = .32, *d* = .25; counting range: *M*_*contr*_ = .77, *SD* = .12, *M*_*dysc*_ = .65, *SD* = .35; *t*(59) = -1.68, *p* = .097, *d* = .43).

**Fig 6:**
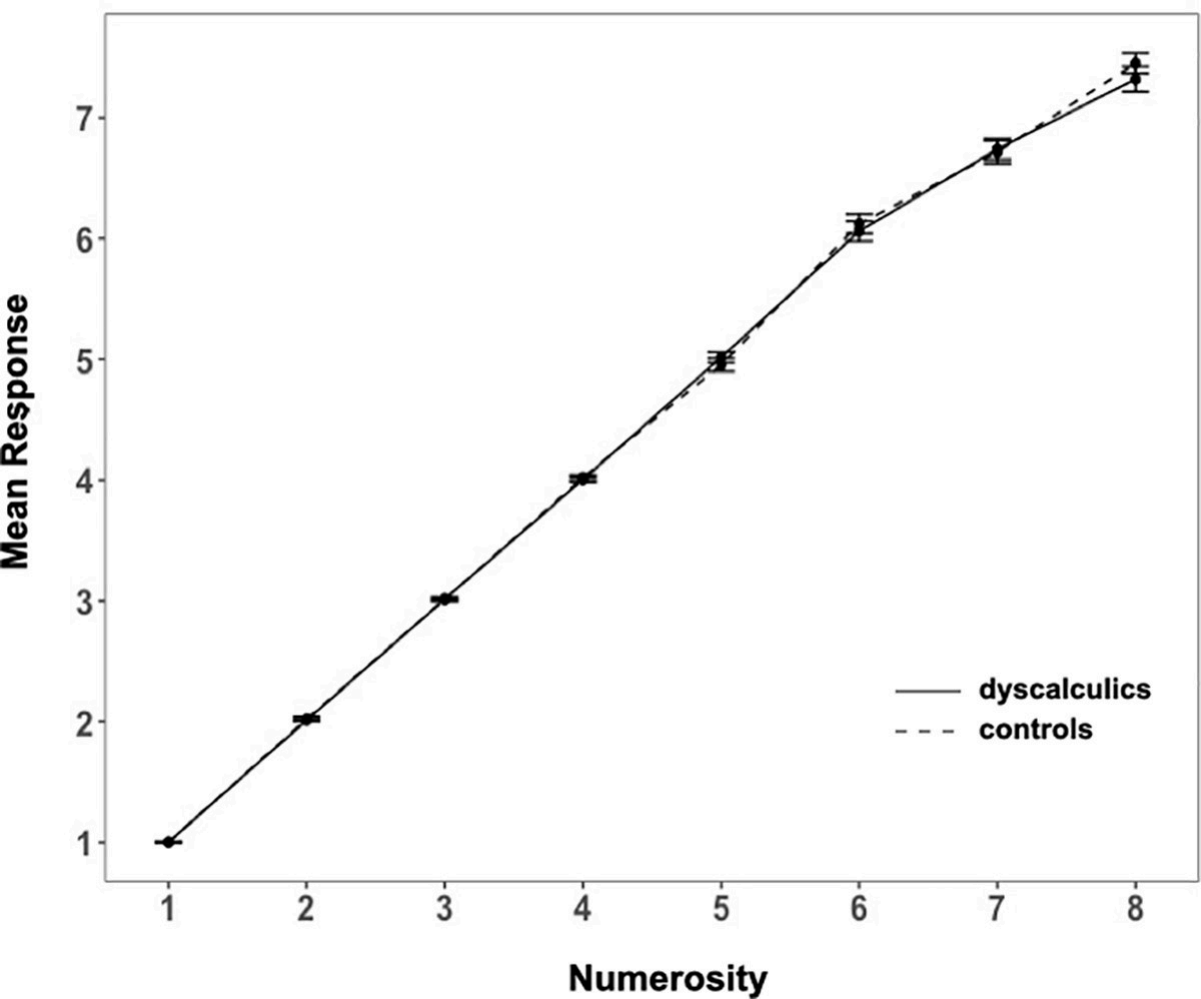
Exact enumeration task: Mean response provided by DD and controls. Error bars represent the standard error of the mean.

The second approach we adopted focused on the response distributions, that we assessed computing the coefficient of variation, providing a quantification of the errors’ extent [29, 40, 67]. This is computed by dividing the standard deviation of the responses by their mean for each numerosity. We found no significant differences between dyscalculics and controls neither in the subitizing range (*M*_*contr*_ = .03, *SD* = .1; *M*_*dysc*_ = .01, *SD* = .06; *p* = .42, *d* = .20) nor in the counting range (*M*_*contr*_ = .13, *SD* = .06; *M*_*dysc*_ = .14, *SD* = .06; *p* = .41, *d* = .21), indicating that for both ranges the size of the errors was similar across groups (see Fig 6).

### Visuo-spatial short-term memory

**Fig 7:**
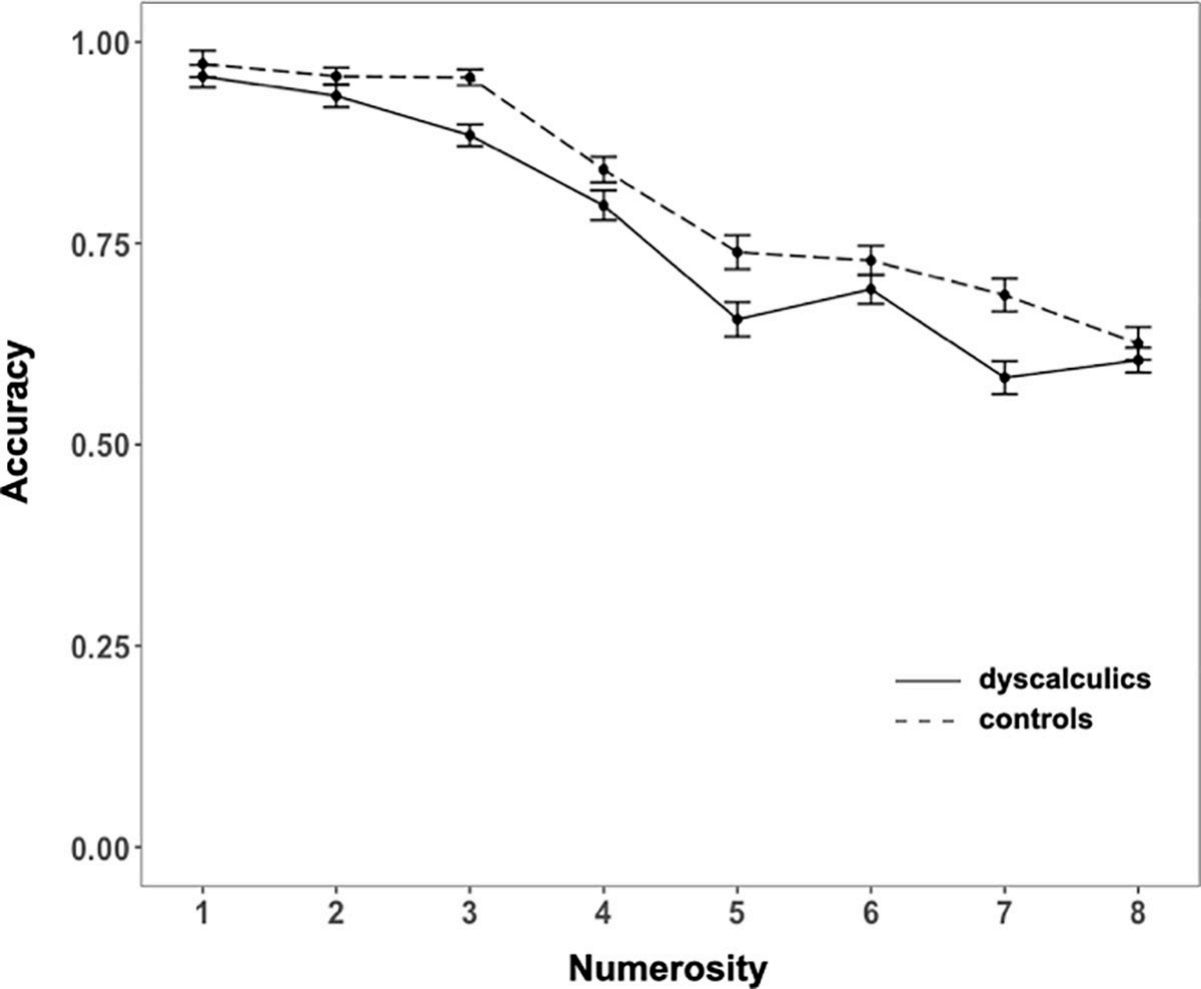
Visuo-spatial short-term memory: accuracy for each numerosity for DD and controls. Error bars represent the standard error of the mean.

We first analyzed mean accuracy through a mixed ANOVA with group as between factor and numerosity as within factor. In both groups accuracy declined with increased set size (set size 1: .96; 2: .94; 3: .92; 4: .82; 5: .69; 6: .71; 7: .63; 8: .61) (main effect of set size: *F*(7, 434) = 173.07, *p* < .001, η^2^_G_ = .66), and dyscalculics performed worse than controls (main effect of group: *F*(1, 62) = 14.3, *p* < .001, η^2^_G_ = .06), especially so for sets of 3 and more items (interaction: *F*(7, 434) = 2.21, *p* = .03, η^2^_G_ = .02) (See Fig 7).

We then calculated Cowan’s K [68] (i.e., K = S(H-F), where S is the set size, H is the hit rate and F is the false alarm rate; the score obtained with this formula allows the estimation of the number of objects encoded) for each set size, and then computed the average K across set sizes for each subject as an estimate of the visuo-spatial short-term memory span. The average K was 1.76 for the dyscalculics (range = .68–2.77, *SD* = .5) and 2.26 for the controls (range = 1.23–3.26, *SD* = .52), a highly significant difference, *t*(62) = -3.85, *p* < .001, *d* = .96.

### Corsi block-tapping test

Separate analyses were performed for Corsi test “forward” and “backward”. Controls performed significantly better than DD, showing a higher visuo-spatial span both forward and backward (“forward”: *M*_*contr*_ = 4.69, *SD* = .96, *M*_*dysc*_ = 4.06, *SD* = .62, *p* = .003, *d* = .77; “backward”: *M*_*contr*_ = 4.59, *SD* = 1.29, *M*_*dysc*_ = 3.12, *SD* = 1.29, *p* < .001, *d* = 1.14).

Because we found impairments in both the ANS acuity and visuo-spatial short-term memory, we addressed the question of the potential link between the two impaired functions by correlating the *w* fraction and accuracy in the ANS task with the Cowan’s K and the average score between the forward and the backward in the Corsi test in DD and controls. We did not find any significant correlation neither in the children with DD, nor in controls (*w* fraction and Cowan’s K: *r*_*contr*_ = -.17, *p* = .39; *r*_*dysc*_ = -.23, *p* = .23; *w* fraction and Corsi test: *r*_*contr*_ = —.13, *p* = .51; *r*_*dysc*_ = .13, *p* = .51; dot comparison accuracy and Cowan’s K: *r*_*contr*_ = —.13, *p* = .49.; *r*_*dysc*_ = .17, *p* = .34; dot comparison accuracy and Corsi test: *r*_*contr*_ = —.13, *p* = .49; *r*_*dysc*_ = .29, *p* = .1). Moreover, we performed the same analyses for the OTS and we did not find significant correlations between subitizing and visuo-spatial short-term memory (accuracy 1–3 and Cowan’s K: *r*_*contr*_ = —.18, *p* = .33; *r*_*dysc*_ = .32, *p* = .07; accuracy 1–3 and Corsi test: *r*_*contr*_ = .26, *p* = .15; *r*_*dysc*_ = .16, *p* = .37; RTs 1–3 and Cowan’s K: *r*_*contr*_ = —.15, *p* = .4.; *r*_*dysc*_ = —.09, *p* = .63; RTs 1–3 and Corsi test: *r*_*contr*_ = .0004, *p* = .99; *r*_*dysc*_ = —.19, *p* = .3).

## Discussion

The main question addressed in our study concerns whether DD is associated with impairments both in the quantification of large sets (supported by the ANS) and of small sets (supported by subitizing, or OTS). Although the dissociation between ANS and OTS is well-established in typically developing infants, young children, and adults, there is currently no agreement on whether the two systems are concurrently impaired in DD. Different theories predict either a defective ANS and a non-impaired OTS [69], or a deficit in both ANS and OTS [70]. Other theories posit that DD results from an impairment in more general cognitive abilities, especially tapping on visuo-spatial skills, such as visuo-spatial working memory [55–57]. In the current study we tested all those three systems (large numerosity comparison, exact enumeration and visuo-spatial short-term memory skills) in the same group of DD children with strong impairments in symbolic number processing and arithmetical calculation (< 2 *SD*s in a standardized mathematical test), and we compared them with a group of children matched for age and general intelligence without deficits in maths.

Regarding the ANS, our study provides strong evidence for a deficit in DD, tightly replicating previous reports [29–32]. DD children showed worse performances compared to typically developing children, resulting in different estimates of their internal Weber fraction, indexing their ANS acuity (dyscalculics: *w* = 0.32; controls: *w* = 0.24). Despite being tested on a different group of DD and controls, who also had a slightly larger age range, it is remarkable that the current findings closely replicate those obtained by Piazza *et al*. [32], who, using the same experimental paradigm and stimuli as used here, reported a *w* of 0.34 in dyscalculics and of 0.25 in controls. Coherent with previous findings, we also observed that although children with DD showed impaired accuracy in this task, they did not present differences in RTs compared to typically developing children. This suggests that, while spending the same time to take the required number comparison decision, dyscalculics rely on less precise internal representations.

Our second skill of interest was subitizing, the ability to accurately enumerate small sets of items. We found no group differences (neither in RTs, in errors, in their combination, nor in error extent) in the subitizing range (1–3). The two groups diverged only in the counting range and only when considering response times, not when considering accuracy.

These data contradict some previous findings [38–42] that reported the presence of a deficit in DD in the subitizing ability. However, an important difference across our study and previous ones in the experimental paradigm might explain the divergent findings: while in our paradigm the dots remained on the screen for a limited amount of time (500 ms), in some of the aforementioned studies children could look at the images and provide their response without time limits. We speculate that this might have prompted the use of divergent strategies (DD using serial exploration, controls using parallel individuation) and that the difference reported does not necessarily reveal a real difference in the underlying skills across groups. Indeed, when children are not allowed to implement serial visual exploration strategies, both RTs and errors performed by children with DD are identical to those of the controls. Moreover, contrary to some of the previous studies, here we defined subitizing range empirically, on the bases of performance in the control group. This is important, as it is well known that the subitizing range depends upon the specificities of the experimental paradigm and stimuli as well as the age of participants. It is possible that those previous studies that set subitizing range a priori erroneously attributed groups difference to subitizing when in fact they might have been related to counting.

Our results of an intact subitizing and impaired large numerosity comparison in DD supports the separation between ANS and multiple-objects individuation system found in typically developing population. These findings lead to confirm a qualitative difference in the mechanisms underlying the perception and representation of large *vs*. small numerosities.

Another relevant finding of the current study was the significant impairment observed in DD in the one-digit symbolic comparison task. In this study we introduced this task because our control group did not perform the diagnostic tests for dyscalculia, and therefore we wanted to have at least one measure of symbolic number processing that would confirm their difference from the DD group. In line with previous studies [34, 71], we found that children with DD performed overall significantly poorly in the symbolic comparison and the difference between the two groups was particularly evident in the case of small distances (1, 2 and 3). This is in line with the idea that the mental representation of numerical symbols in DD is less precise (such that close numbers are less differentiated from each other) than in controls. The fact that DD children present impairments in symbolic comparison is part of the predictions put forward by many authors, not only those that ascribe DD to an impaired number sense that would later affect the symbolic number processing [32, 72] but also those who postulate a first deficit in the exact numerical representation of symbolic numbers with a later and consequent dysfunction in the ANS acuity [73]. The contrast between these two positions is still debated in the literature and the question of the possible causal/effect link between symbolic and non-symbolic number processing remains open.

Finally, our results also confirmed previous reports [46] that children with DD are also heavily impaired in visuo-spatial short-term memory, which we measured both through a computerized change detection task and the Corsi test, a widely used test in neuropsychological assessment. In the change detection task, we found that children with DD retain less information of a complex visual scene (they have a smaller Cowan’s K). Similar results were obtained in the Corsi test where children had to repeat (“forward” or “backward”) a sequence of previously shown touched blocks. This task required not only encoding/storage/retrieval of the information but also its mental manipulation, in particular in the “backward” version. In both versions of the tasks, children with DD were dramatically less accurate compared to the controls. Importantly, however, we found that deficits in those visuo-spatial tasks unlikely account for the impairments in the ANS as the two did not correlate across subjects. It is possible that impairments in visuo-spatial skills and in the ANS play a differential role in numeracy: while the former may potentially have a higher impact on learning and using written and mental arithmetical calculation procedures [74, 75], the latter may be more fundamental for learning and using the semantics of symbolic numbers.

Previous work [10] on adult participants found that subitizing and visuo-spatial short-term memory are correlated as they share common limited attentional resources. From this, one would predict that the two abilities should be commonly impaired (or commonly spared) in DD. However, we found that subitizing was fully spared and visuo-spatial short-term memory deeply impaired. It is hard to speculate on why there is this apparent discrepancy with the adult results. One possibility is that the two functions are unrelated in childhood but they get related during development. This issue needs more studies directly comparing different age ranges using the same experimental paradigms.

In sum, the present study reports a strong and independent weakness of dyscalculics in two different cognitive domains: approximate number processing (ANS) and visuo-spatial processing. The absence of correlations in performance across the two domains suggests that the impairments in the ANS may not be determined by general impairments in visuo-spatial processing, and that the two functions are separable even if they are concurrently impaired in DD.

The present study failed to observe weaknesses in subitizing, thus failing to provide evidence for the subitizing deficit hypothesis in DD. We speculate that the contrasting findings with previous studies could be explained by methodological differences. It would be important that the field converges on set of stable methods used universally to investigate the ANS and subitizing, such that the results will be more directly comparable.

### Implications for practice

If further replicated, these results lead to important clinical implications: on one side they suggest that clinicians could increase symbolic abilities since we found strong impairments in the capacity to compare digits, also in line with previous data showing a strong link between symbolic number knowledge and maths skills. Clinicians could also combine exercises that aim at strengthening the ANS acuity especially using non-symbolic approximate calculation [76–78; note however that this issue remains controversial as some authors questioned the effectiveness of the ANS training and others suggested that ANS training studies lack of sufficient statistical power; see 79–81]. More generally, combining the results of the present study with those in the literature, we think that it is reasonable to define DD as connected to multiple deficits rather than a single core deficit [82–84]. From the point of view of the potential clinical implications, this work suggests that training for dyscalculics should also include exercises aiming at boosting visuo-spatial skills.
